# External hydrocephalus in adults: an insidious cause of delayed intracranial hypertension. Report of 33 relevant cases treated with success by CSF lumbar drainage

**DOI:** 10.1186/2045-8118-12-S1-P31

**Published:** 2015-09-18

**Authors:** Romain Manet, Romain Guerin, Orianne Martinez, Gilles Francony, Jean-Paul Roustan, Jean-François Payen, Serge Molliex, Jêrome Morel, Laurent Gergele

**Affiliations:** 1 Department of Neurosurgery, University hospital of Saint-Etienne, France; 2 Department of Intensive Care, University hospital of Grenoble, France; 3 Department of Intensive Care, University hospital of Montpellier, France; 4 Department of Intensive Care, University hospital of Saint-Etienne, France

## Introduction

The concept of external hydrocephalus refers to situations of CSF flow impairment within subarachnoid spaces (SAS). Classically described in infant and children, literature offers few data on adult.

## Material and methods

We retrospectively analysed adult patients admitted in four French intensive care units, between November 2010 and December 2014, for severe traumatic brain injury (TBI) or subarachnoid hemorrhage (SAH). We undertook clinical and radiological findings of patients presenting intracranial hypertension (ICHT) presumably in relation with external hydrocephalus (delayed ICHT concomitant to a paradoxical enlargement of subarachnoid spaces), treated with cerebrospinal fluid (CSF) external lumbar drainage (ELD).

## Results

33 patients (19 men, mean age 46.7 yrs [+/-17.5]) admitted for TBI (n=22), SAH (n=8) or other brain insults (n=3) with a mean initial Glasgow score of 8 (+/- 4) were included. 25 (75.8%) patients did not receive former external ventricular drainage. In all cases, ELD was dramatically effective to lower intracranial pressure (25.2 mmHg [+/-9.1] before EDL vs 7.4 mmHg [+/-6.0] after EDL). No mydriasis or intracranial bleeding occurred. One patient (3%) developed an ELD infection. Patients were discharged from ICU with a mean modified Rankin Score of 4[+/-1].

## Conclusions

Often described as a passive process (e.g. hygroma), CSF accumulation around the brain after acute cerebral insults in adults can be approached as an active process of external hydrocephalus. This diagnosis remains often subtle, but should systematically be evoked when CT scan show paradoxical enlargement of subarachnoid spaces in a context of ICHT. Our data tend to confirm that in these specific situations, ELD should be considered as a safe, effective and minimal invasive option.

